# Rosmarinic Acid Targets AKR1B1 to Ameliorate Atherosclerosis via Vascular Endothelial Cell Energy Metabolism Regulation

**DOI:** 10.3390/biom16030403

**Published:** 2026-03-09

**Authors:** Taoli Sun, Quanye Luo, Tingting Liu, Xuzhen Lv, Limei Lin, Duanfang Liao, Qinhui Tuo, Wen Chen

**Affiliations:** 1 Key Research Laboratory of Germplasm Resources and Standardized Planting of Genuine Regional Medicinal Materials Produced in Hunan Province, School of Pharmacy, Hunan University of Chinese Medicine, Changsha 410208, China; suntaoli@stu.hnucm.edu.cn (T.S.); 20223777@stu.hnucm.edu.cn (Q.L.); 20242085@stu.hnucm.edu.cn (T.L.); 20232054@stu.hnucm.edu.cn (X.L.); limei_lin@hnucm.edu.cn (L.L.); dfliao@hnctcm.edu.cn (D.L.); 2 School of Pharmacy, Hunan University of Medicine, Huaihua 418000, China; 3 Key Laboratory of Vascular Biology and Translational Medicine, Medical School, Hunan University of Chinese Medicine, Changsha 410208, China

**Keywords:** rosmarinic acid, atherosclerosis, energy metabolism, endothelial cells, AKR1B1

## Abstract

Atherosclerosis (AS), a chronic cardiovascular disease, originates from endothelial dysfunction, a process closely linked to cellular energy metabolism. While rosmarinic acid (RA) exhibits protective cardiovascular effects, its precise mechanism against AS remains undefined. This study demonstrates that RA alleviates AS in ApoE^−/−^ mice, as evidenced by reduced aortic plaques, enhanced CD31 expression, and improved serum NO and ET-levels. Integrating network pharmacology and experimental validation, we identified Aldo-keto reductase family 1 member B1 (AKR1B1) as a direct functional target of RA. Mechanistically, RA downregulated AKR1B1, thereby activating the SIRT3/PFKFB3 axis. In Ox-LDL-induced HUVECs, RA enhanced viability, reduced ROS, and boosted energy metabolism, indicated by elevated ECAR, OCR, and levels of G-6-P, F-6-P, and ATP. Crucially, RA rescued endothelial injury induced by AKR1B1 overexpression via this pathway. Our findings establish that RA protects against AS by directly targeting AKR1B1 to restore endothelial energy homeostasis through the AKR1B1/SIRT3/PFKFB3 signaling axis, offering a novel therapeutic strategy.

## 1. Introduction

Atherosclerosis (AS) is a chronic, progressive disease characterized by the accumulation of lipid plaques within the arterial walls. These plaques are composed chiefly of cholesterol, lipids, and inflammatory cells, leading to the hardening and narrowing of arteries, resulting in cardiovascular events such as heart attacks and strokes [1,2]. Atherosclerotic cardiovascular disease is a leading cause of mortality worldwide, resulting in millions of deaths each year [3,4]. It is characterized by endothelial dysfunction, vascular inflammation, and the buildup of atheromatous plaques in medium and large arteries [5].

A key initiating mechanism in AS development is endothelial dysfunction. Injured vascular endothelial cells release inflammatory mediators such as tumor necrosis factor-alpha (TNF-α), interleukin-1 beta (IL-1β), and adhesion molecules, which trigger inflammation and recruit more inflammatory cells to the vessel wall. This initiates a vicious cycle of inflammation, promoting vascular smooth muscle cell proliferation, plaque formation, and oxidative stress [6]. This pathological process is closely linked to cellular energy metabolism. Disorders in energy metabolism result in inadequate ATP supply, impair cell function, and induce oxidative stress by generating excessive reactive oxygen species (ROS), thereby directly damaging endothelial cells [7,8]. Hence, protecting endothelial cells and maintaining their proper function is crucial for preventing the onset and progression of AS. Given the critical role of energy metabolism in endothelial homeostasis, therapeutic strategies targeting metabolic pathways may offer new avenues for AS treatment [9].

Medication plays a central role in AS treatment, but current drugs often have significant drawbacks. For example, statins, which are commonly used for lipid-lowering, can cause liver and kidney damage as well as rhabdomyolysis [10]. Similarly, antiplatelet drugs, used to prevent blood clots, can lead to gastrointestinal discomfort and bleeding [11]. Therefore, identifying novel and safer therapeutic agents is crucial. In this context, compounds from medicinal plants have garnered significant interest for their potential anti-atherosclerotic properties [12,13].

Rosmarinic acid (RA) is a natural polyphenolic compound found in a variety of medicinal plants, including *Rosmarinus officinalis*, *Salvia officinalis*, and *Perilla frutescens*. Its chemical structure is (2R)-3-(3,4-dihydroxyphenyl)-2-[(E)-3-(3,4-dihydroxyphenyl) prop-2-enoyl] oxypropanoic acid. RA is a potent anti-inflammatory and antioxidant agent, making it a popular ingredient in cosmetic products and food [14]. Many studies have shown that RA can prevent cardiovascular disease, offering benefits such as lowering plasma cholesterol, improving vascular function, reducing vascular inflammation, and providing anti-thrombotic effects [15,16,17]. Pharmacokinetically, RA exhibits limited oral absorption (~1% of administered dose) and undergoes extensive metabolism by gut microbiota into absorbable phenolic acids. Following absorption, RA is rapidly distributed via binding to serum albumin and undergoes phase II conjugation reactions in the liver and intestine, with saturation kinetics observed at higher doses. Renal excretion represents the primary elimination pathway, with most metabolites cleared within 24 h. Importantly, clinical studies have reported no serious adverse effects associated with RA-containing extracts, supporting its favorable safety profile for long-term applications [18]. Although RA has been reported to exhibit endothelial protective effects, its specific function and mechanism in treating AS through the protection of endothelial cells remain unclear.

Previous studies have provided valuable insights into RA’s protective mechanisms, suggesting it can suppress endothelial inflammation by inhibiting the ROS/p38/FOXO1/TXNIP pathway under hyperglycemic conditions [19] and alleviate oxidative stress-induced dysfunction via AMPK/eNOS signaling [20]. However, whether RA can directly target a specific protein to regulate the fundamental process of endothelial energy metabolism in atherosclerosis remains unexplored. In this study, we employed ApoE^−/−^ mice to assess the protective effects of RA against AS in vivo. Furthermore, we investigated the role of RA in endothelial cell protection and its potential interaction with cellular energy metabolism pathways. This research aims to elucidate the mechanisms underlying RA’s preventive potential against AS.

## 2. Materials and Methods

### 2.1. Animal Protocol

Ten male-specific pathogen-free (SPF) C57BL/6 mice (6–8 weeks old) and 40 male apolipoprotein E-deficient (ApoE^−/−^) mice (6–8 weeks old) were procured from the Laboratory Animal Center, Hunan University of Chinese Medicine (Changsha, China). The mice were acclimatized in a controlled environment for one week before the experiment. C57BL/6 mice were fed a standard diet, while ApoE^−/−^ mice received a high-fat diet (HFD, 77.5% basal diet, 20% lard, 2% cholesterol, and 0.5% sodium cholate) for 12 weeks, ad libitum access to food and water. All experimental techniques followed the guidelines for the Care and Use of Laboratory Animals and were conducted in compliance with the ARRIVE guidelines 2.0.

The study included five groups with 10 mice each. The control group (Ctrl) consisted of C57BL/6 mice given normal saline. 40 ApoE^−/−^ mice were randomly assigned to four groups: the model group (HFD, saline), a low-dose RA group (RA_L_, 10 mg/kg/day), a high-dose RA group (RA_H_, 20 mg/kg/day), and the atorvastatin group (AT, 10 mg/kg/day). Treatments were administered orally at 0.1 mL per 10 g body weight using gastric gavage after four weeks of feeding, continuing for eight weeks. Mice were weighed weekly and monitored for general health.

RA (C_18_H_16_O_8_, CAS 20283-92-5, purity > 98%) was acquired from Yuanye Bio-Technology Co., Ltd. (Shanghai, China) and AT from Hangzhou MSD Pharmaceutical Co., Ltd. (Hangzhou, China). Both were suspended in normal saline, with final concentrations of 1.0 mg/mL for RA_L_ and AT and 2.0 mg/mL for RA_H_. The doses of RA were selected based on our previous study showing that *Prunella vulgaris* polyphenols (containing approximately 10% RA) at 100 and 200 mg/kg exerted anti-atherosclerotic effects [21], which was further validated by our preliminary dose-ranging experiments and supported by published literature.

At the end of the experiment, animals were anesthetized with 50 µg/g pentobarbital (China National Medicines Co. Ltd., Beijing, China) before collecting blood samples and the entire aorta.

### 2.2. Oil Red O (ORO) Staining

ORO staining was utilized to visualize lipid accumulation and localization in the aorta [22]. Briefly, aortic tissue was fixed in 4% paraformaldehyde for 72 h. Longitudinal incisions were made to expose the intimal surface of the aorta, and lesions were detected using 0.5% ORO solution (Sigma-Aldrich, Shanghai, China). Additionally, frozen cross-sections of the aortic root, arch, and abdominal aorta were stained with 0.5% ORO solution. Plaque areas were quantified using Image-Pro Plus 6.0 and GraphPad Prism 9.0 software.

### 2.3. Immunohistochemical Analysis [22]

Paraffin-embedded cross sections of aortic root tissues were prepared using a microtome. Sections were incubated overnight at 4 °C with anti-CD31 antibody (1:100, AF3628, Novus Biologicals, Littleton, CO, USA). After washing with phosphate balanced solution (PBS), sections were treated with a secondary antibody (1:500, Pinuofei Biotechnology, Wuhan, China) for 30 min at room temperature, followed by hematoxylin counterstaining. Images were captured with a microscope (Motic Electrical Technology, Xiamen, China), and CD31 quantification was performed using Image-Pro Plus 6.0 software.

### 2.4. Detection of Serum Nitric Oxide (NO) and Endothelin-1 (ET-1) Levels

Collected blood samples were centrifuged at 3000 r/min for 15 min to separate serum. NO levels were measured using the NO assay kit (Griess Reagent, S0021S, Beyotime Biotechnology, Shanghai, China), and ET-1 levels were measured using the ET-1 assay kit (E-EL-M2730C, Elabscience Biotechnology, Wuhan, China).

### 2.5. Collects of Drug and Disease Related Targets [23]

TCMSP, BATMAN-TCM (a score cutoff of 20 and altered *p*-value of 0.05), and SwissTargetPrediction (probability > 0) databases were used to predict the protein targets of RA. Targets related to AS and EC protection were identified from PharmGKB, Therapeutic Target Database (TTD), and DrugBank. All target protein names were converted to official gene symbols using the UniProt database. The intersecting targets for RA, AS, and EC protection were analyzed using a Venn diagram tool (Summary of all online database URLs in Table 1).

### 2.6. Kyoto Encyclopedia of Genes and Genomes (KEGG) Enrichment Analysis

R 4.3.0 software was used to convert the intersecting target gene symbols to Entrez IDs, followed by KEGG pathway enrichment analysis. Pathways with *Q* values below 0.05 were considered statistically significant [23].

### 2.7. Constructs Protein–Protein Interaction (PPI) Network

The PPI network for the intersecting target genes was constructed using the STRING database (with the species set to “*Homo sapiens*”). A minimum interaction score of 0.90 was applied to ensure the reliability of the predicted interactions [23].

### 2.8. Molecular Docking

Molecular docking was performed with AutoDock Vina 1.5.6 [24] to predict protein-ligand interactions, following procedures from previous research [23]. First, the 3D structures of AKR1B1 (PDB: 8FH5) and RA (Pubchem CID: 5281792) were prepared and converted to pdbqt format. Then, interactions between AKR1B1 and RA were analyzed. Finally, PyMOL 2024 software was utilized to visualize the interactions, with hydrogen bonds highlighted by black dashed lines [23].

### 2.9. Molecular Dynamics Simulation

After the docking process, molecular dynamics (MD) simulations were employed to assess the stability of the AKR1B1-RA complex following the approach outlined in our previous research [23]. The system was modeled using the AmberFF99SB force field for the protein and the GAFF force field for RA. The complex was solvated in a cubic water box with a 1 nm buffer, and Na^+^ ions were added to neutralize the system.

The simulation workflow included: (1) Energy minimization: A two-step minimization was carried out. First, the protein was restrained while water molecules were minimized (1500 steps of steepest descent, total 5000 cycles). Then, the entire system was minimized without restraints (2000 steps of steepest descent, total 5000 cycles). (2) Equilibration: The system was gradually heated from 0 to 310 K over 100 ps using the Langevin thermostat, followed by 100 ps of pressure equilibration at 1 bar using the isotropic Berendsen coupling method. (3) Production run: A 100 ns MD simulation was conducted at a constant temperature of 310 K (human body temperature) and a pressure of 1 bar, with an integration time step of 2 fs. The cutoff distance for van der Waals and short-range electrostatic interactions was set to 10 Å, and long-range electrostatics were treated using the Particle-Mesh-Ewald (PME) method.

All simulations were performed using GROMACS (version 2018 or later). Trajectory analysis, including root-mean-square deviation (RMSD) and root-mean-square fluctuation (RMSF), was performed using GROMACS built-in tools, and binding modes were visualized with PyMOL 2024. The stability of the AKR1B1-RA complex was assessed throughout the 100 ns trajectory, and the representative structure was extracted for further analysis.

### 2.10. Cell Culture

Primary human umbilical vein endothelial cells (HUVECs) were provided by Meisen Cell Technology Co., Ltd. (CTCC-0804-PC, Hangzhou, China). All cells were maintained in an endothelial cell-specific culture medium (ECM) (Meisen, Hangzhou, China) enriched with 5% fetal bovine serum (FBS), 1% endothelial cell growth factor human (ECGS), and 1% penicillin-streptomycin (P/S). Cells were cultured at 37 °C in a CO_2_-incubated atmosphere, and passage three to eight were used for experiments. HUVECs were chosen as they are a well-established model for investigating vascular endothelial cell function and signaling pathways.

### 2.11. Cellular Thermal Shift Assay (CETSA) [25]

HUVECs were seeded into 10 cm dishes at a density of 5.0 × 10^6^ cells/dish for incubating 48-h incubation. The samples were then processed following the CETSA protocol. Briefly, (1) Cells were washed with ice-cold PBS twice and collected in 1.5 mL EP tubes. (2) The cell suspension was immersed in liquid nitrogen for 40 s, followed by immediate thawing in a 37 °C water bath. This process was repeated twice. (3) Samples were centrifuged at 20,000 g at 4 °C for 15 min, and the supernatant was collected. (4) RA was added at varying concentrations (0.3, 1, 3, 10, 30, 100, 300, 1000 μM), or gradient temperatures were applied to the protein samples, followed by Western blot analysis.

### 2.12. Activity Measurement of AKR1B1

The Aldose Reductase Activity (Colorimetric) assay kit (ab273276, Abcam, Cambridge, UK) was used to measure the activity of AKR1B1. All steps, including enzyme solution preparation, substrate preparation, reaction condition setting, reaction mixture preparation, reaction monitoring, and data analysis for AKR1B1 enzyme kinetics determination were strictly conducted according to the instructions of the kit.

### 2.13. Surface Plasmon Resonance (SPR) Detection [26]

The SPR technology was used to detect the affinity of AKR1B1 and RA. The activator is prepared by mixing 400 mM 1-ethyl-3-(3-dimethylaminopropyl)-carbodiimide (EDC) and 100 mM N-hydroxysuccinimide immediately prior to injection. The CM5 sensor chip is activated for 420 s with the mixture at a flow rate of 10 μL/min. Dilute AKR1B1 to 50 μg/mL in immobilization buffer, then injected to sample channel (Fc4) at a flow rate of 10 μL/min, and typically result in immobilization levels of 6000 RU, the reference channel (Fc1) does not need ligand immobilization step. The chip is deactivated by 1 M Ethanolamine hydrochloride at a flow rate of 10 μL/min for 420 s. Dilute RA with the same analyte buffer to 7 concentrations (3125, 1562.5, 780, 390, 195, 97.5 and 0 nM). RA is injected to channel Fc1- Fc4 at a flow rate of 10 μL/min for an association phase of 120 s, followed by 200 s dissociation. The association and dissociation process are all handling in the analyte buffer. Repeat 7 cycles of analyte according to analyte concentrations in ascending order. After each cycle of interaction analysis, the analyte will dissociate naturally.

### 2.14. Immunofluorescence Staining

Aortic arch tissue sections were deparaffinized by immersion in xylene, followed by a graded series of ethanol washes (100%, 95%, 85%, 60%) to remove paraffin wax and prepare the fixed, dehydrated tissue for staining. After antigen retrieval, the sections were rinsed with 0.3% Triton X-100 for 10 min and blocked with 5% bovine serum albumin at 37 °C for one hour. The sections were then incubated with primary antibodies, including goat anti-CD31 (1:100, AF3628, Novus Biologicals, USA), rabbit anti-AKR1B1 (1:500, A22132, Abclonal Technology, Wuhan, China), rabbit anti-SIRT3 (1:100, ab189860, Abcam, UK), and rabbit anti-PFKFB3 (1:100, ab181861, Abcam, UK) at 4 °C for 48 h. After washing with PBS, the sections were treated with Alexa Fluor-488 donkey anti-rabbit IgG (1:1000, ab150073, Abcam, UK) and Alexa Fluor-594 donkey anti-goat IgG (1:1000, ab150132, Abcam, UK) for two hours at 37 °C in the dark. Sections were then stained with 4′,6-diamidino-2-phenylindole (DAPI) (F6057, Sigma, St. Louis, MO, USA) and mounted with coverslips. Images were captured utilizing a Nikon A1R HD25 confocal microscope (Nikon, Shinagawa, Tokyo, Japan), and the fluorescence images were analyzed with Fiji-ImageJ software.

### 2.15. Cell Counting Kit-8 (CCK-8) Assay

Cell viability of HUVECs was tested by CCK-8 Kit (BS350B, Biosharp, Changsha, China). HUVECs were seeded into 96-well plates and cultured for 24 h until 70–80% confluence. Cells were exposed to varying concentrations of RA (0–10 µM) for 48 h to assess toxicity. In another set of experiments, cells were pretreated with RA (0.3, 1, 3 µM) for one hour, followed by exposure to 100 µg/mL oxidized low-density lipoprotein (Ox-LDL) for an additional 48 h to determine RA’s protective effect on ECs. After the treatment period, cells were incubated with ECM containing 10% CCK-8 reagent for one hour, and absorbance at 450 nm was monitored utilizing a microplate reader.

### 2.16. Reactive Oxygen Species (ROS) Detection

HUVECs were seeded into 6-well plates at a density of 5.0 × 10^5^ cells/well and grown to 70%–80% confluence. Cells were pretreated with RA (0.3, 1, 3 µM) for one hour, followed by treatment with 100 µg/mL Ox-LDL for an additional 48 h. After incubation, cells were washed twice with PBS and ROS levels were detected using the DCFH-DA (2′,7′-Dichlorofluorescin diacetate) method, as per the instructions of the ROS assay Kit (S0175, Beyotime Biotechnology, Shanghai, China). ROS signals were quantified utilizing ImageJ 1.8.0 software.

### 2.17. Measurement of Cellular Energy Metabolism

To assess the effects of RA on energy metabolism in HUVECs, glycolysis and mitochondrial function were measured. Cells were seeded onto a cell energy metabolism culture plate at a density of 2.5 × 10^3^ per well for 24 h incubation at 37 °C in a CO_2_ atmosphere. After being pretreated with drugs according to the experimental design, a Seahorse XFp analyzer (Angilent, Santa Clara, CA, USA) was used to detect extracellular acidification rate (ECAR) and oxygen consumption rate (OCR), following the instructions of the ECAR (103346-100, Angilent, Santa Clara, CA, USA) and OCR (103010-100, Angilent, USA) assay kits.

### 2.18. Targeted Energy Metabolomics

HUVECs were seeded into 10 cm cell culture dishes at a density of 5.0 × 10^6^ cells/dish and incubated for 24 h. After reaching 70–80% confluency, the cells were treated with 3 μM RA and 100 μg/mL Ox-LDL according to the experimental design. Cell samples were separated via the 1290 Infinity LC ultra-high-performance liquid chromatography system (UHPLC), followed by analysis with a 5500 QTRAP mass spectrometer in negative ion mode.

### 2.19. Nicotinamide Adenine Dinucleotide (NAD^+^/NADH) Assay

After seeding HUVECs into 6 cm cell culture dishes at a density of 2.0 × 10^6^ cells/dish and achieving a density of 70–80%, the cells were pretreated with 3 µM RA for one hour, followed by treatment with 100 µg/mL Ox-LDL for an additional 48 h. After incubation, cells were washed twice, and NAD^+^/NADH levels were evaluated using the NAD^+^/NADH assay kit (S0175, Beyotime Biotechnology, Shanghai, China).

### 2.20. Lentiviral Transduction

*Akr1b1* overexpression was achieved through lentiviral transduction. AKR1B1 recombinant lentivirus (pLV[Exp]-CMV>AKR1B1 [NM_001628.4]-EF1A>GFP:T2A, 2.80×10^8^ TU/mL) and empty vector lentivirus (pLV[Exp]-CMV>MSC-EF1A>GFP:T2A, 3.77×10^8^ TU/mL) were obtained from HyCyte^®^ Co., Ltd. (Nanjing, China). After transduction, cells were harvested for further experiments 48 h post-transduction.

### 2.21. Real-Time Quantitative PCR (RT-qPCR) Analysis

Total RNAs were isolated using TRIzol reagent (15596026, Invitrogen, Carlsbad, CA, USA) and quantified by Nanodrop2000 (Thermo Fisher Scientific, Waltham, MA, USA). Then, the obtained RNAs were reverse-transcribed into cDNA using 2×Taq Master Mix (P111-01, Vazyme, Nanjing, China) on T100 TM Thermal Cycler (Bio-Rad, Hercules, CA, USA). RT-qPCR was carried out with cDNA in triplicate utilizing ChamQ Universal SYBR qPCR Master Mix (Q711-02, Vazyme, China) on a real-time PCR system (LightCycler^®^ 96, Roche Life Science, Indianapolis, IN, USA). Specific quantitative primer sequences are provided in Table 2.

### 2.22. ATP Content Determination

HUVECs were seeded in 6 cm cell culture dishes at a density of 2.0 × 10^6^ cells/dish and achieving a density of 70–80%, the cells were pretreated with 3 µM RA for one hour, followed by treatment with 100 µg/mL Ox-LDL for an additional 48 h. After incubation, cells were washed twice, and ATP levels were evaluated using the ATP chemiluminescence assay kit (E-BC-F002, Elabscience, Wuhan, China).

### 2.23. Western Blot Analysis

Protein samples were prepared with radio-immunoprecipitation assay (RIPA) buffer (BOSTER, Biological Technology, Wuhan, China) supplemented with 0.1% protease inhibitor and 0.1% phosphatase inhibitor. Total protein concentrations were measured using a bicinchoninic acid assay (BCA) kit (E-BC-K318-M, Elabscience Biotechnology Co., Ltd., Wuhan, China). Proteins were then separated by sodium dodecyl sulfate-polyacrylamide gel electrophoresis (SDS-PAGE) (CW2384, CWBIO, Taizhou, China) on an 8–10% gradient gel, followed by the transfer to a polyvinylidene difluoride (PVDF) membranes (Merck-Millipore, Burlington, MA, USA). The membranes were blocked with skim milk for one hour at 37 °C and incubated overnight at 4 °C with the following primary antibodies: anti-AKR1B1 (A22132, 1:5000, Abclonal Technology, Wuhan, China), anti-SIRT3 (ab189860, 1:1000, Abcam, Cambridge, UK), anti-PFKFB3 (ab181861, 1:1000, Abcam, Cambridge, UK), anti-β-tubulin (10068-1-AP, Proteintech, Wuhan, China), and anti-β-actin (20536-1-AP, Proteintech, Wuhan, China). Subsequently, the membranes were incubated for one hour with goat anti-mouse (1:10000, Proteintech, Wuhan, China) and goat anti-rabbit (1:10000, Proteintech, Wuhan, China) at 37 °C. In the end, the membranes were visualized utilizing enhanced chemiluminescence (ECL) horseradish peroxidase (HRP) substrate (BL520B, Biosharp, Changsha, China) followed by autoradiography (ChemiDoc XPS + System, Bio-rad, Hercules, USA). Band intensity was analyzed with Image Lab.

### 2.24. Data Analysis

GraphPad Prism version 9.0 was employed to statistically analyze data (expressed as means ± S.E.M.). Measures were analyzed utilizing a *t*-test or One-way analysis of variance (ANOVA), depending on the dataset. *p* value < 0.05 was defined as significant.

## 3. Results

### 3.1. RA Mitigates HFD-Induced Atherosclerotic Plaque Development in ApoE^-/-^ Mice

**Figure 1:**
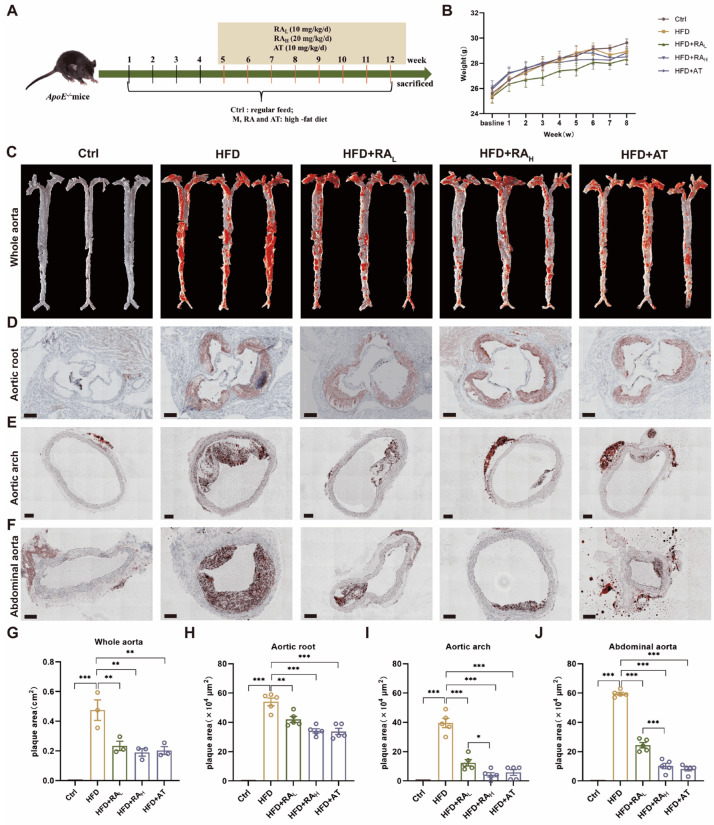
RA attenuates atherosclerotic lesion development in ApoE^−/−^ mice fed with HFD. (**A**) Schematic diagram of the experimental design. (**B**) Body weight comparison across treatment groups (n = 10). (**C**) Representative images of ORO staining in whole aortas (n = 3). (**D**–**F**) Representative cryosection images of ORO staining in the aortic root (scale bar: 200 μm), aortic arch (scale bar: 100 μm), and abdominal aorta (scale bar: 100 μm), respectively (n = 5). (**G**) Quantification of plaque area in whole aortas (n = 3). (**H**–**J**) Quantification of plaque area in the aortic root, aortic arch, and abdominal aorta, respectively (n = 5). RA: rosmarinic acid; AT: atorvastatin; Ctrl: control; HFD: high-fat diet; HFD + RA_L_: HFD + 10 mg/kg/day RA; HFD + RA_H_: HFD + 20 mg/kg/day RA; HFD + AT: HFD + 10 mg/kg/day AT. Data are presented as mean ± S.E.M., * *p* < 0.05, ** *p* < 0.01, *** *p* < 0.001.

The therapeutic efficacy of RA in treating AS was investigated using ApoE^-/-^ mice fed with HFD. All treatment groups, including AT as a positive control, were shown in the experiment design (Figure 1A). Body weight comparisons revealed no significant differences among the various experimental groups (Figure 1B). ORO staining was performed to assess lipid deposition in the vasculature. In the Ctrl group, blood vessels remained smooth with no visible lipid deposition on the vascular intima, aortic roots, arches, or abdominal aortas. In contrast, the HFD group exhibited significant plaque-like lipid deposits throughout the aorta, indicating the successful establishment of the AS model. Treatment with RA, as well as AT, mitigated the effects of HFD on lipid deposition in whole aorta (Figure 1C,G). Both RA and AT groups showed reduced plaque formation in the aortic root, aortic arch, and abdominal aorta compared to the HFD group (Figure 1D–F,H–J). High-dose RA (RA_H_) significantly reduced aortic root and abdominal aorta plaque formation compared to low-dose RA (RA_L_) (Figure 1E,F,I,J). These results demonstrate that RA can reduce atherosclerotic lesions in ApoE^−/−^ mice, suggesting its therapeutic value in the treatment of AS.

### 3.2. RA Attenuates High-Fat Diet-Induced Endothelial Damage in ApoE^−/−^ Mice

**Figure 2:**
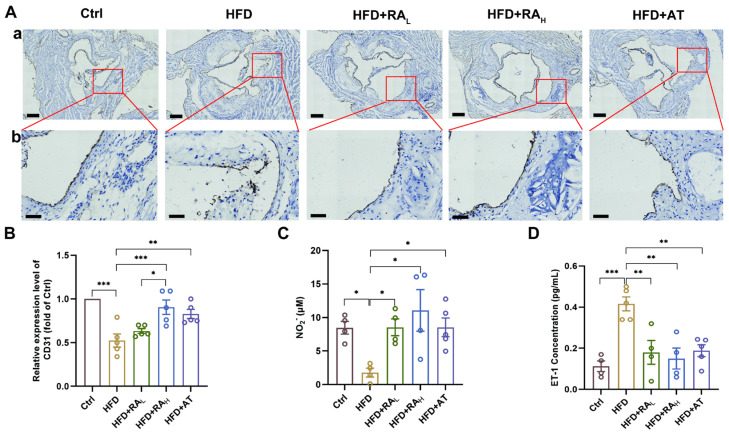
RA protects the vascular endothelium in ApoE^−/−^ mice. (**A**) Representative images of immunohistochemical staining for CD31 in the aortic root; (**a**) Low magnification, scale bar: 200 μm; (**b**) High magnification, scale bar: 50 μm (n = 5). (**B**) Quantification of relative CD31 expression levels in the aortic root (n = 5). (**C**,**D**) Serum levels of NO and ET-1, respectively (n = 4–5). RA: rosmarinic acid; AT: atorvastatin; Ctrl: control; HFD: high-fat diet; HFD + RA_L_: HFD + 10 mg/kg/day RA; HFD + RA_H_: HFD + 20 mg/kg/d RA; HFD + AT: HFD + 10 mg/kg/d AT; NO, nitric oxide; ET-1, endothelin-1. Data are presented as mean ± S.E.M., * *p* < 0.05, ** *p* < 0.01, *** *p* < 0.001.

CD31, a highly glycosylated Ig-like membrane receptor, is consistently expressed on the vascular endothelium [27]. In atherosclerotic disease, CD31 plays a critical role in limiting collateral inflammatory damage and maintaining endothelial barrier function [28]. To assess the effect of RA on endothelial integrity, we evaluated CD31 expression through immunohistochemical staining. As shown in Figure 2A,B, the CD31 level in the HFD group was significantly lower than in the Ctrl group, which is consistent with findings from previous studies [29,30]. Notably, RA_H_ and AT treatments significantly increased CD31 expression, while RA_L_ treatment did not, indicating that higher doses of RA might be necessary to preserve endothelial integrity.

Nitric oxide (NO), a soluble gas synthesized from L-arginine in endothelial cells, is essential for vascular homeostasis and endothelial function [31]. Conversely, endothelin-1 (ET-1), a potent vasoconstrictor peptide released by the endothelium, can impair endothelial cell function through dysregulation of vascular tone [32]. To further investigate the protective effects of RA on the vascular endothelium, serum levels of NO_2_^−^ and ET-1 were measured and analyzed. As shown in Figure 2C, the concentration of serum NO_2_^−^ was significantly decreased in the HFD group compared to the Ctrl group, indicating impaired NO production. Notably, treatment with RA_L_, RA_H_, or AT significantly increased NO_2_^−^ concentration compared to the HFD group, suggesting a restoration of endothelial function. In contrast, serum ET-1 levels were elevated in the HFD group, reflecting endothelial dysfunction. However, treatment with RA_L_, RA_H_, or AT significantly reduced ET-1 levels (Figure 2D), further supporting the protective effects of RA on the vascular endothelium.

### 3.3. RA Targets AKR1B1 to Regulate Endothelial Energy Metabolism in AS

**Figure 3:**
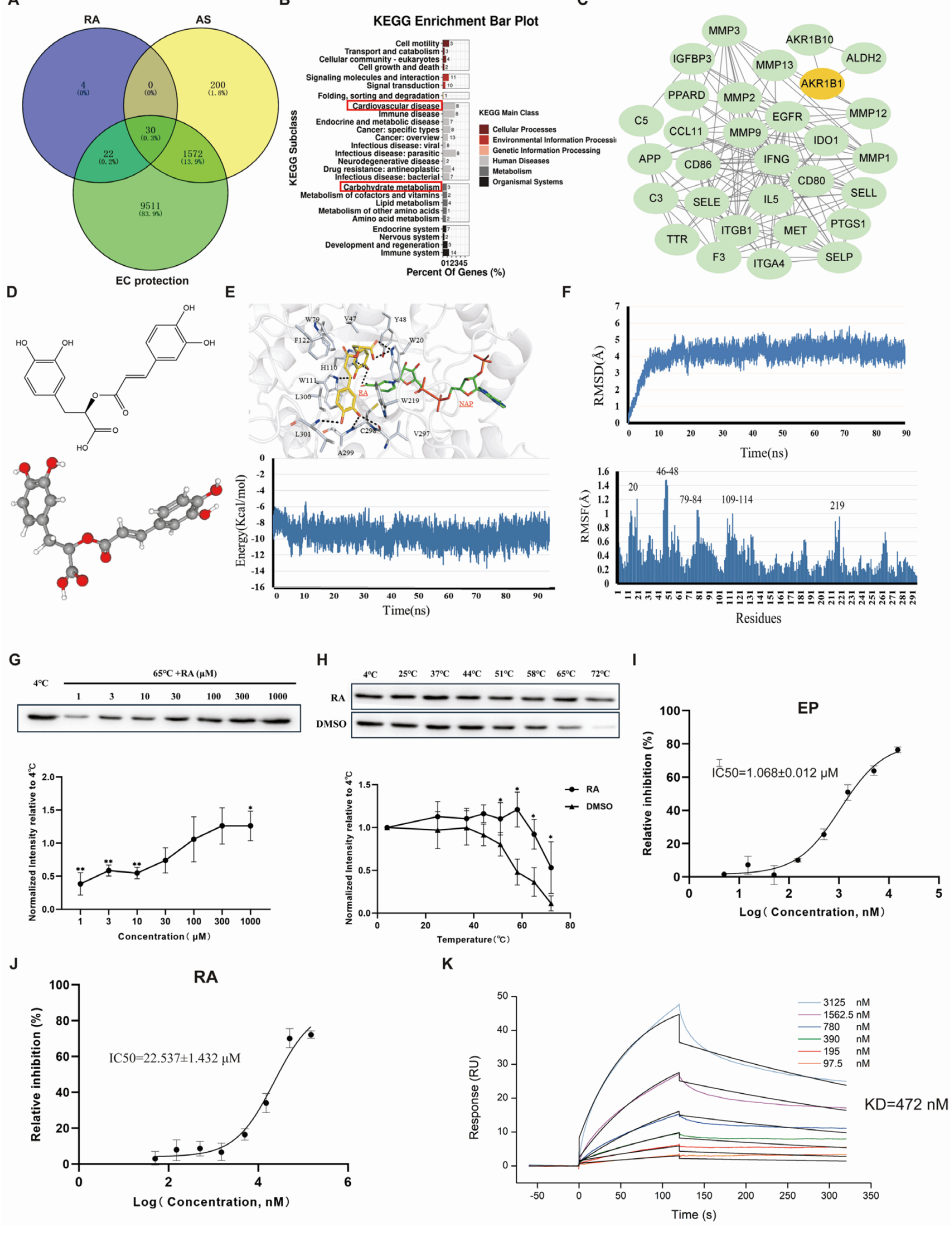
Prediction and validation of RA’s anti-atherosclerotic targets and underlying mechanisms in endothelial protection. (**A**) Venn diagram showing the intersection of RA targets, atherosclerosis AS-associated genes, and EC protection-related genes. (**B**) KEGG pathway analysis of the intersecting target genes. (**C**) Protein–protein interaction (PPI) network of the intersecting target genes. (**D**) 2D and 3D structures of RA. (**E**) Molecular docking model and total energy of the AKR1B1-RA complex as a function of time. (**F**) Root-mean-square deviation (RMSD) of the AKR1B1 backbone with RA and the average root-mean-square fluctuation (RMSF) value of each atom of RA when in complexation with AKR1B1. (**G**,**H**) Cellular thermal shift assay (CETSA) demonstrating the interaction between RA and AKR1B1. The original WB images are shown in Figure S1. (**I**,**J**) Inhibitory effects of EP and RA on AKR1B1 activity. (**K**) The affinity of between AKR1B1 and RA. RA, rosmarinic acid; AS, atherosclerosis; EC, endothelial cell; AKR1B1, aldo-keto reductase family 1, member B1; EP: epalrestat. Data are presented as mean ± S.E.M., * *p* < 0.05, ** *p* < 0.01.

The study next investigated the comprehensive mechanism and key target of RA in the treatment of AS, we used a combination of network pharmacology analysis, molecular docking, molecular dynamics simulation, CETSA, and enzyme kinetics determination. As shown in Figure 3A, a Venn diagram identified 56 RA-related target genes, 1802 AS-related target genes, and 11,135 endothelial cell protection-related target genes after removing duplicates. Subsequently, 30 intersecting target genes were identified, as listed in Table 3.

KEGG enrichment analysis of the 30 intersecting target genes revealed that RA likely acts against AS primarily through pathways implicated in cardiovascular diseases and, most notably, through carbohydrate metabolism (Figure 3B). This suggests that RA protects endothelial cells by modulating energy production pathways [33]. We focused on whether RA’s anti-AS effects were mediated through its regulation of endothelial energy metabolism.

The PPI network of the 30 intersecting targets was generated using STRING and then imported into Cytoscape 3.8.2 for visualization (Figure 3C). Among the targets, EGFR, MMP9, and ITGB1 had higher degree values, indicating a strong correlation with AS development [34,35]. However, AKR1B1, AKR1B10, and ALDH2 were primarily linked to energy metabolism [36,37]. Notably, AKR1B1 acted as a bridge connecting EGFR, MMP9, and ITGB1 with AKR1B10 and ALDH2 in the PPI network. Based on these findings, we propose that AKR1B1 is a key target through which RA exerts its anti-AS effects by regulating EC energy metabolism.

Molecular docking results revealed a strong interaction between RA and AKR1B1, with RA forming hydrogen bonds with key residues W20, H110, W111, L301, and C298 in AKR1B1. The binding energy remained stable at approximately −9.3 kcal/mol from 10 to 90 ns, indicating a high affinity between RA and AKR1B1 (Figure 3D,E). Molecular dynamics simulation further explored the binding stability between RA and AKR1B1. As illustrated in the RMSD graph (Figure 3F), the system exhibited fluctuations between 0 and 10 ns, suggesting initial instability as the protein adjusts to RA binding. From 10 to 90 ns, the RMSD value stabilized, with a mean value of around 4.24 Å during the production phase, indicating that the system reached a stable conformation. Additionally, RMSF analysis showed that residues W20, Y47, Y48, W79, H110, and W111 had fluctuations exceeding 1.0 Å, suggesting significant interaction at these sites. Overall, these results suggested that the RA-AKR1B1 complex is highly stable in an aqueous environment, supporting the robustness of their binding interaction.

To investigate the direct interaction between RA and AKR1B1 in vitro, dose- and temperature-dependent CETSA were conducted [38]. As depicted in Figure 3G, at 65 °C, increasing concentrations of RA enhanced AKR1B1 stability and 300 μM RA was chosen for temperature-dependent CETSA. Subsequently, 300 μM RA or DMSO was incubated with AKR1B1-containing cell lysates for three minutes at 4 °C, 25 °C, 37 °C, 44 °C, 51 °C, 58 °C, 65 °C, and 72 °C followed by protein immunoblot. RA-treated cell lysates showed significant protein stabilization from 4 °C through 65 °C. In contrast, DMSO-treated cell lysates showed more AKR1B1 degradation at 51 °C, 58 °C, 65 °C, and 72 °C (Figure 3H).

To evaluate RA’s inhibitory effect on AKR1B1, aldose reductase activity was measured by monitoring nicotinamide adenine dinucleotide phosphate (NADPH) reduction. Epalrestat (EP), a known AKR1B1 inhibitor, was used as a positive control. At concentrations ranging from 0.1 to 300 μM, EP inhibited AKR1B1 with an IC50 of 1.068 ± 0.012 µM (Figure 3I), whereas RA showed a higher IC50 of 22.537 ±1.432 µM (Figure 3J). SPR analysis demonstrated that AKR1B1 and RA exhibit a high binding affinity with a dissociation constant (KD) of 472 nM, as shown in Figure 3K. These results confirm that RA directly interacts with AKR1B1 and suggest that RA may function as an inhibitor of AKR1B1 activity.

### 3.4. RA Modulates AKR1B1, SIRT3, and PFKFB3 Expression in the Aortic Arch Intima of ApoE^−/−^ Mice Fed with HFD

**Figure 4:**
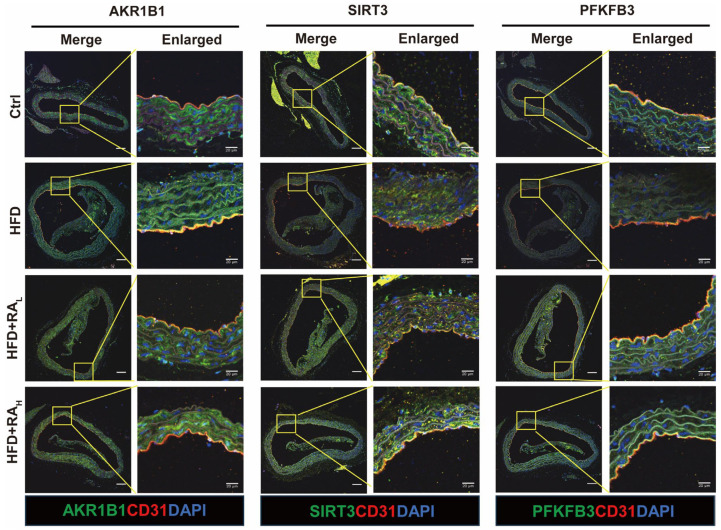
RA modulates the expression of AKR1B1, SIRT3, and PFKFB3 in the aortic arch intima of ApoE^−/−^ mice. Representative immunofluorescence images showing the expression levels of AKR1B1, SIRT3, and PFKFB3 (green) in the ECs (labeled with CD31, red) of the aortic arch intima. RA treatment reduced AKR1B1 expression while increasing SIRT3 and PFKFB3 levels compared to the HFD group. Scale bar: 100 μm.

Although it has been established that RA can directly interact with AKR1B1, the downstream molecular mechanisms through which RA regulates energy metabolism and exerts its protective effects on the vascular endothelium via AKR1B1 remain to be elucidated. AKR1B1, a key enzyme in the polyol pathway, catalyzes the conversion of glucose to sorbitol, which is subsequently converted to fructose by sorbitol dehydrogenase. This process consumes NAD^+^, a crucial electron carrier and signaling molecule in eukaryotic cells [39,40]. SIRT3 (Sirtuin 3), a NAD^+^-dependent deacetylase, plays a vital role in maintaining mitochondrial balance and regulating glycolytic metabolism in cardiovascular diseases [41,42]. In particular, SIRT3 deficiency in mice leads to reduced expression of the glycolytic enzyme PFKFB3 (6-phosphofructo-2-kinase/fructose-2,6-biphosphatase 3) in ECs, resulting in impaired glycolysis [43]. Therefore, AKR1B1 activation may modulate the downstream proteins SIRT3 and PFKFB3, ultimately affecting EC energy metabolism. We employed immunofluorescence staining to examine the expression levels of AKR1B1, SIRT3, and PFKFB3 in the aortic arch across different experimental groups (Figure 4). Compared to Ctrl group, the HFD group exhibited significantly elevated AKR1B1 expression, while both SIRT3 and PFKFB3 levels were notably reduced. Remarkably, RA treatment effectively reduced AKR1B1 expression while simultaneously increasing SIRT3 and PFKFB3 levels in the aortic arch intima of ApoE^−/−^ mice. These findings suggest that prolonged HFD consumption can lead to impaired energy metabolism in the aortic arch intima. RA exerts its protective effects on vascular ECs by modulating the expression of key proteins involved in energy metabolism pathways.

### 3.5. RA Protects HUVECs from Ox-LDL-Induced Damage by Modulating the AKR1B1/SIRT3/PFKFB3 Axis and Reducing Oxidative Stress

**Figure 5:**
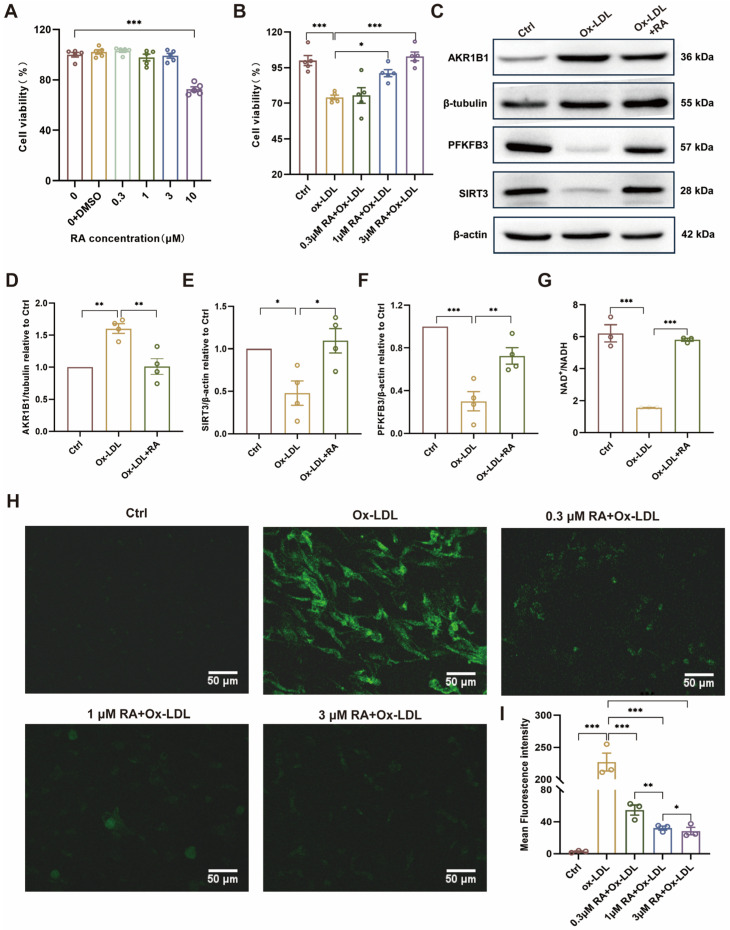
RA attenuated Ox-LDL-induced injury by AKR1B1/SIRT3/PFKFB3 axis in HUVECs. (**A**,**B**) CCK-8 assay results for cell viability. (**C**) Representative Western blot detection image of AKR1B1, PFKFB3, and SIRT3 protein levels in HUVECs, respectively (n = 4). The original WB images are shown in Figure S2. (**D**–**F**) Semi-quantitative analysis of AKR1B1, PFKFB3, and SIRT3 protein level in HUVECs, respectively (n = 4). (**G**) NAD^+^/NADH level determined by ELISA. (**H**) Detection of ROS fluorescence by DCFH-DA. (**I**) Semi-quantitative analysis of ROS fluorescence intensity level in HUVECs. RA, rosmarinic acid; DMSO, dimethyl sulfoxide; Ctrl: Control; Ox-LDL: oxidized low-density lipoprotein; Ox-LDL + RA; Ox-LDL-induced HUVECs pretreated with RA; ROS: reactive oxygen species. Data are presented as mean ± S.E.M., * *p* < 0.05, ** *p* < 0.01, *** *p* < 0.001.

To further validate the protective effects of RA observed in the animal experiments, its potential toxicity and protective effects were investigated at the cellular level using HUVECs. Consistent with the in vivo findings, cell viability assays revealed that RA concentrations between 0.3 and 3 μM had no significant toxicity (Figure 5A). Moreover, pretreatment with RA at 1 and 3 µM significantly attenuated the decrease in cell viability caused by Ox-LDL exposure (Figure 5B). These findings suggested that RA has a favorable safety profile and can protect HUVECs from Ox-LDL-induced damage. To elucidate the underlying mechanisms of RA’s protective effects, the investigation centered on the AKR1B1/SIRT3/PFKFB3 axis. Western blot analysis demonstrated that RA treatment inhibited the Ox-LDL-induced upregulation of AKR1B1 and restored the protein levels of SIRT3 and PFKFB3 (Figure 5C–F). AKR1B1, a key enzyme in the polyol pathway, its upregulation leads to a decrease in the NAD^+^/NADH ratio. Ox-LDL treatment lowered NAD^+^/NADH levels, likely due to AKR1B1 overexpression. However, RA treatment effectively restored the NAD^+^/NADH ratio (Figure 5G), indicating its ability to maintain cellular energy homeostasis via the AKR1B1/SIRT3/PFKFB3 axis.

Furthermore, the effects of RA on Ox-LDL-induced ROS production in HUVECs were investigated by using a fluorescent probe, DCFH-DA. Confocal microscopy revealed that untreated cells (Ctrl) exhibited minimal ROS levels, while Ox-LDL-treated cells showed significantly elevated ROS production. Pretreatment with RA dose-dependently reduced the Ox-LDL-induced ROS intensity from 0.3 to 1 μM (Figure 5H,I), confirming its antioxidant properties and protective effects against Ox-LDL-induced oxidative stress in HUVECs. These findings highlight the potential therapeutic value of RA in preventing or treating endothelial dysfunction associated with AS.

### 3.6. RA Improves Cellular Energy Metabolism in Ox-LDL-Treated HUVECs

**Figure 6:**
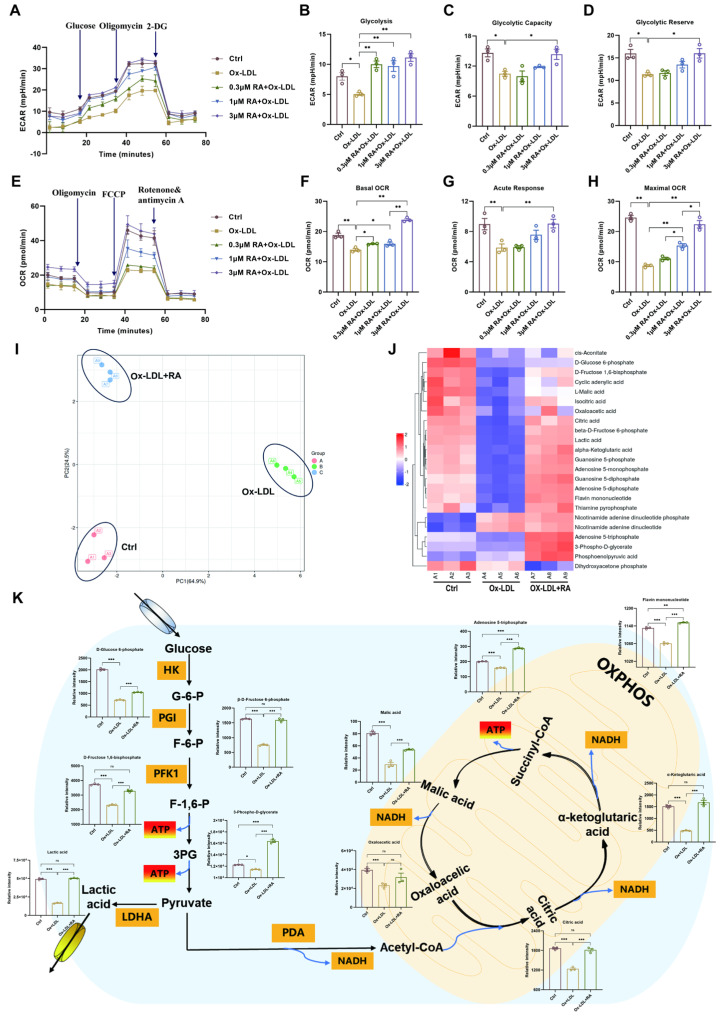
RA improves cellular energy metabolism in Ox-LDL-treated HUVECs. (**A**) ECAR, a measure of glycolytic function, was quantified in real-time using a Seahorse XF analyzer. (**B**–**D**) Quantification of glycolysis, glycolytic capacity, and glycolytic reserve in HUVECs (n = 3). (**E**) OCR, a measure of mitochondrial respiration, was quantified in real-time using a Seahorse XF analyzer. (**F**–**H**) Quantification of basal OCR, acute response, and maximal respiration in HUVECs (n = 3). (**I**) Principal component analysis (PCA) of metabolites after RA treatment (concentration of RA: 3 μM). (**J**) Heatmap of targeted energy metabolites in Ctrl, Ox-LDL, and Ox-LDL + RA groups. (**K**) Changes in the content of differentially expressed metabolites in the energy metabolism pathway after RA treatment. Ctrl: control; Ox-LDL: oxidized low-density lipoprotein; RA: rosmarinic acid; ECAR: extracellular acidification rate; OCR: oxygen consumption rate; 2-DG: 2-Deoxy-D-Glucose; FCCP, Carbonyl cyanide 4-(trifluoromethoxy) phenylhydrazone; G-6-P: D-Glucose 6-phosphate; F-6-P: β-D-Fructose 6-phosphate; F-1,6-P: D-Frutose 1,6-bisphosphate; 3PG: 3-phospho-D-glycerate; HK: hexokinase; PGI: glucose 6-phosphate isomerase; PFK1: phosphofructokinase-1; ATP: adenosine triphosphate; LDHA: lactate dehydrogenase A; PDA: pyruvate dehydrogenase; NADH: nicotinamide adenine dinucleotide; OXPHOS: oxidative phosphorylation; Data are present as mean ± S.E.M., * *p* < 0.05, ** *p* < 0.01, *** *p* < 0.001, ns means no statistically significant difference.

To further investigate the impact of RA on Ox-LDL-induced alterations in energy metabolism, extracellular acidification rate (ECAR) and oxygen consumption rate (OCR) measurements were performed on HUVECs. Cells were pretreated with various concentrations of RA (0.3, 1, 3 μM) for one hour, followed by exposure to 100 μg/mL Ox-LDL for 48 h. Measurements were conducted using the Seahorse XFp energy metabolism analyzer. As shown in Figure 6A,E, Ox-LDL treatment resulted in significant decreases in ECAR and OCR compared to the ctrl group, indicating impaired energy metabolism. However, pretreatment with RA, most notably at 3 μM, effectively reversed these detrimental effects, improving glycolysis, glycolytic capacity, glycolytic reserve, basal OCR, acute response, and maximal OCR in Ox-LDL-treated cells (Figure 6B–D,F–H). These findings suggest that RA can protect ECs from Ox-LDL-induced metabolic dysfunction by restoring energy metabolism.

To gain a more comprehensive understanding of the metabolic changes induced by RA, targeted energy metabolomics was employed to explore the impact of 3 μM RA on energy metabolites in HUVECs (Figure 6). Principal component analysis (PCA) revealed distinct differences among the control, Ox-LDL, and Ox-LDL + RA groups (Figure 6I). A total of 23 metabolites were obtained using a 5500 QTRAP mass spectrometer, and a heatmap was generated using TBtools v2.225 software (Figure 6J). Key energy metabolites were then mapped to the energy metabolism pathway (Figure 6K). Ox-LDL treatment led to a downregulation of several crucial metabolites, including D-Glucose 6-phosphate (G-6-P), β-D-Fructose 6-phosphate (F-6-P), D-Fructose 1,6-bisphosphate (F-1,6-P), 3-phospho-D-glycerate (3PG), lactic acid, citric acid, α-ketoglutaric acid, adenosine 5-triphosphate (ATP), malic acid, oxaloacetic acid, and flavin mononucleotide, compared to the Ctrl group. Remarkably, pretreatment with RA effectively reversed these changes, leading to an upregulation of these metabolites in the Ox-LDL + RA group compared to the Ox-LDL group.

The ECAR and OCR measurements, along with the targeted energy metabolomics data, provide compelling evidence that RA can protect endothelial cells from Ox-LDL-induced metabolic dysfunction by modulating key energy metabolites and restoring energy metabolism.

### 3.7. AKR1B1 Overexpression Downregulates SIRT3 and PFKFB3, Leading to Impaired Glycolytic Function and Mitochondrial Respiration in HUVECs

**Figure 7:**
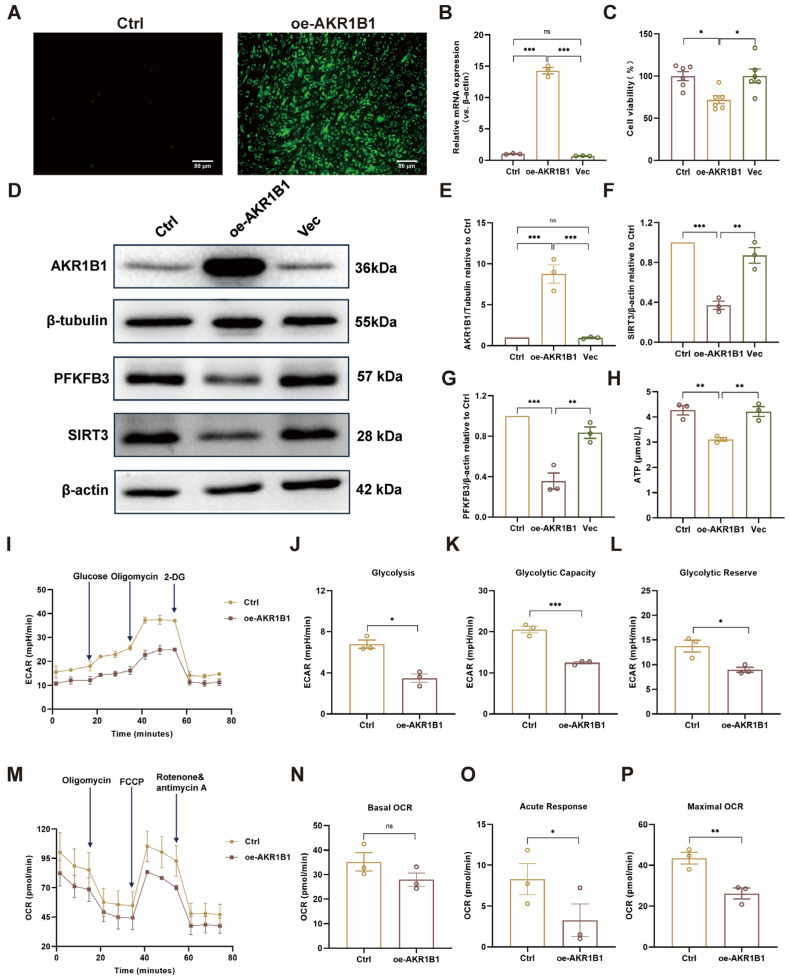
Overexpression of AKR1B1 in HUVECs impairs cellular energy metabolism. (**A**) Fluorescence microscopy confirms AKR1B1 overexpression in lentivirus-transduced HUVECs. (**B**) Quantitative RT-PCR analysis of AKR1B1 mRNA expression in HUVECs (n = 3). (**C**) Cell viability in HUVECs after AKR1B1 overexpression. (**D**) Western blot analysis of AKR1B1, SIRT3, and PFKFB3 protein levels in HUVECs. The original WB images are shown in Figure S3. (**E**–**G**) Semi-quantitative analysis of AKR1B1, SIRT3, and PFKFB3 protein levels in HUVECs, respectively (n = 3). (**H**) ATP levels in HUVECs after AKR1B1 overexpression. (**I**) ECAR in Ctrl and oe-AKR1B1 HUVECs was quantified in real-time using a Seahorse XF analyzer. (**J**–**L**) Quantification of glycolysis, glycolytic capacity, and glycolytic reserve in Ctrl and oe-AKR1B1 HUVECs (n = 3). (**M**) OCR in Ctrl and oe-AKR1B1 HUVECs was quantified in real-time using a Seahorse XF analyzer. (**N**–**P**) Quantification of basal OCR, acute response, and maximal respiration in Ctrl and oe-AKR1B1 HUVECs (n = 3). Ctrl: control; oe-AKR1B1: overexpression of AKR1B1 in HUVECs; Vec: vector; ECAR: extracellular acidification rate; OCR: oxygen consumption rate; 2-DG: 2-Deoxy-D-Glucose; FCCP, Carbonyl cyanide 4-(trifluoromethoxy) phenylhydrazone. Data are presented as mean ± S.E.M., * *p* < 0.05, ** *p* < 0.01, *** *p* < 0.001, ns means no statistically significant difference.

Evidence from animal and cell experiments suggests that RA might protect the vascular endothelium through the AKR1B1/SIRT3/PFKFB3 signaling axis. To further investigate this potential mechanism and verify whether AKR1B1 directly regulates SIRT3 and PFKFB3 in vascular ECs, an AKR1B1-overexpressing HUVEC line (oe-AKR1B1) was established. Fluorescence microscopy revealed that the oe-AKR1B1 group exhibited significantly stronger green fluorescence compared to the Ctrl group, confirming successful lentivirus transduction in cells (Figure 7A). RT-qPCR results demonstrated significantly increased AKR1B1 mRNA levels in oe-AKR1B1 cells compared to the Ctrl and vector (Vec) groups (Figure 7B). Furthermore, AKR1B1 overexpression reduced cell viability in HUVECs, suggesting that elevated AKR1B1 expression may contribute to endothelial cell dysfunction (Figure 7C). Western blot analysis further confirmed elevated AKR1B1 protein expression, validating the successful establishment of an AKR1B1 overexpression cell model. Obviously, overexpression of AKR1B1 led to significantly reduced expression levels of SIRT3 and PFKFB3 (Figure 7D–G), suggesting that these two proteins might be downstream targets of AKR1B1. This finding is consistent with our previous results, which indicated that RA might exert its protective effects on the vascular endothelium by modulating the AKR1B1/SIRT3/PFKFB3 signaling axis.

To further investigate the impact of AKR1B1 overexpression on energy metabolism, an ATP chemiluminescence assay kit along with a Seahorse XFp energy metabolism analyzer was employed to measure intracellular ATP, ECAR and OCR levels in HUVECs. As shown in Figure 7H–P, oe-AKR1B1 cells exhibited significantly reduced ATP, ECAR and OCR compared to the Ctrl group cells. Notably, glycolysis, glycolytic capacity, glycolytic reserve, acute response, and maximal OCR were all substantially decreased in oe-AKR1B1 cells. These findings suggest that AKR1B1 overexpression impairs both glycolytic function and mitochondrial respiration in HUVECs, which consequently leads to a reduction in cellular ATP levels. Taken together, these results provide compelling evidence that AKR1B1 directly regulates SIRT3 and PFKFB3 in vascular ECs, and that overexpression of AKR1B1 leads to impaired energy metabolism, likely through the downregulation of these downstream targets.

### 3.8. RA Treatment Ameliorates AKR1B1 Overexpression-Induced EC Dysfunction In Vitro

**Figure 8:**
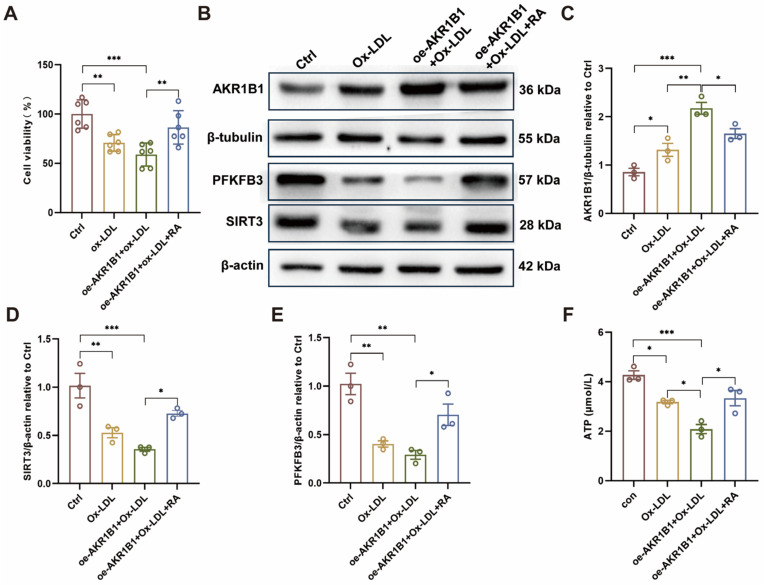
RA administration rescues AKR1B1 overexpression-induced EC injury in vitro. (**A**) Cell viability assay in HUVECs. (**B**) Representative Western blot images showing the protein levels of AKR1B1, SIRT3, PFKFB3, β-tubulin and β-actin in HUVECs under different conditions. The original WB images are shown in Figure S4. (**C**–**E**) Semi-quantitative analysis of AKR1B1 (**C**), SIRT3 (**D**), and PFKFB3 (**E**) protein levels relative to β-tubulin and β-actin in HUVECs (n = 3). (**F**) Cellular ATP levels in HUVECs following AKR1B1 overexpression and RA treatment. Ctrl: control; Ox-LDL: oxidized low-density lipoprotein; RA: rosmarinic acid; oe-AKR1B1: overexpression of AKR1B1 in HUVECs; Data are presented as mean ± S.E.M., * *p* < 0.05, ** *p* < 0.01, *** *p* < 0.001.

The results demonstrate the protective effects of RA on AKR1B1 overexpression-induced EC dysfunction in vitro. As shown in Figure 8A, oe-AKR1B1 significantly reduced cell viability compared to the Ctrl. However, RA treatment (oe-AKR1B1+Ox-LDL+RA) partially rescued the decrease in cell viability caused by AKR1B1 overexpression, suggesting that RA can help maintain EC survival and function under stress conditions.

Western blot analysis (Figure 8B–E) revealed that AKR1B1 overexpression downregulated the protein levels of SIRT3 and PFKFB3, two key metabolic regulators in ECs. Importantly, RA treatment partially restored the expression of both SIRT3 and PFKFB3 in oe-AKR1B1 cells, indicating that RA can counteract the detrimental effects of AKR1B1 overexpression on cellular metabolism. Furthermore, as shown in Figure 8F, AKR1B1 overexpression significantly reduced ATP levels in ECs compared to the Ctrl group. This decrease in ATP levels was partially attenuated by RA treatment, suggesting that RA can help maintain cellular energy homeostasis in ECs under conditions of metabolic stress induced by AKR1B1 overexpression.

## 4. Discussion

The predominant energy source for endothelial cells is glycolysis, with over 85% of ATP production relying on this pathway, even under aerobic conditions [44]. The distinct metabolic phenotype of endothelial cells highlights the pivotal importance of glycolytic homeostasis in preserving vascular function, with its disruption recognized as a critical mechanism in the development of AS [45]. Elevated glycolytic activity has been implicated in promoting angiogenesis, which may destabilize atherosclerotic plaques [46]. Conversely, a moderate level of glycolysis plays a protective role in mitigating endothelial injury, positioning it as a promising therapeutic target for AS [45].

Our network pharmacology approach predicted that the energy metabolism pathway is the primary regulatory mechanism of RA in AS, with aldose reductase (AKR1B1) identified as the key target. AKR1B1 serves as the rate-limiting enzyme in the polyol pathway. Under pathological states such as inflammation or oxidative stress, AKR1B1 is activated, catalyzing the conversion of glucose to sorbitol. This process reduces intracellular glucose availability and consumes NAD^+^, thereby suppressing glycolysis and disrupting cellular energy metabolism [47,48]. Our research confirms that RA directly binds to AKR1B1, as validated by cellular thermal shift assay (CETSA) and surface plasmon resonance (SPR). Enzyme kinetics assays further show that RA reduces AKR1B1′s activity, supporting the hypothesis that RA targets AKR1B1 to regulate EC energy metabolism. Enzyme kinetics assays revealed that RA inhibits AKR1B1 with an IC_50_ of 22.5 µM, notably higher than epalrestat (1.07 µM). This difference is likely due to distinct binding modes: molecular docking shows RA interacts with residues W20, H110, W111, L301, and C298, whereas EP binds to a different set. Importantly, RA also downregulates AKR1B1 protein expression in vitro and in vivo, indicating a dual regulatory mechanism that may enhance its functional impact beyond the IC_50_ value.

The downstream consequences of AKR1B1 inhibition involve critical metabolic regulators. The consumption of NAD^+^ by the activated polyol pathway [39,40]. NAD^+^ serves as both an electron carrier in eukaryotic cell metabolism and a participant in cellular signal transduction through its utilization by enzymes such as sirtuins [41]. Sirtuin 3 (SIRT3), a prominent NAD^+^-dependent deacetylase, is critical for maintaining mitochondrial balance and metabolism, including the regulation of glycolytic metabolism in cardiovascular diseases [42]. Notably, SIRT3 knockout in mice reduces the expression of the glycolytic enzyme 6-phosphofructo-2-kinase/fructose-2,6-bisphosphatase 3 (PFKFB3) in endothelial cells, leading to decreased glycolysis [43]. Therefore, SIRT3 and PFKFB3 are potentially downstream proteins modulated by AKR1B1 in endothelial cell energy metabolism. Our results align with this axis: in ApoE^−/−^ mice, AKR1B1 expression was significantly increased in the aortic arch intima, while SIRT3 and PFKFB3 levels were reduced. RA treatment effectively downregulated AKR1B1 and upregulated SIRT3 and PFKFB3. These findings suggest that RA, by targeting AKR1B1, influences the downstream SIRT3/PFKFB3 pathway to modulate endothelial cell energy metabolism.

The protective role of RA on endothelial cells was consistent across cellular and animal models. Targeted energy metabolomic analysis revealed that glycolysis and mitochondrial respiration products were decreased in Ox-LDL-induced HUVECs but were upregulated upon RA treatment, providing direct evidence of RA’s impact on endothelial cell energy metabolism. Previous research has established that disruptions in EC energy metabolism can impair vascular function and contribute to vascular pathologies [49]. Crucially, energy metabolism pathways, such as glycolysis and mitochondrial respiration, are critical for energy production and maintaining redox homeostasis [50,51]. To functionally validate the pivotal role of AKR1B1 in this process, we overexpressed AKR1B1 in HUVECs and observed a significant reduction in ECAR, OCR, and ATP levels—confirming that AKR1B1 activation disrupts cellular energy homeostasis. Importantly, RA treatment effectively rescued this metabolic impairment induced by AKR1B1 overexpression. Together, these functional data reinforce that RA regulates endothelial cell energy metabolism primarily through the AKR1B1/SIRT3/PFKFB3 axis.

Several phenolic acids, such as salvianolic acid A, cinnamic acid, and caffeic acid, have been reported to exhibit vascular protective properties [52,53,54]. Among these, RA, a hydrophobic phenolic acid mainly found in Lamiaceae plant species, has been shown to inhibit diabetic endothelial dysfunction by blocking inflammasome activation in endothelial cells [55]. Our study extends these findings by elucidating a novel, metabolism-focused mechanism for RA in the context of AS-associated endothelial dysfunction, moving beyond its known anti-inflammatory effects.

While this study provides compelling evidence for the role of RA in regulating endothelial cell energy metabolism through the AKR1B1/SIRT3/PFKFB3 signaling axis, there are some limitations to be addressed in future research. First, although HUVECs are a well-established and widely used in vitro model for investigating endothelial cell function, they do not fully recapitulate the complex hemodynamic environment and cellular heterogeneity of the arterial endothelium in vivo. Future studies employing more advanced models, such as microfluidic chips or primary arterial endothelial cells, may provide additional insights. Second, our in vivo experiments were conducted exclusively using male ApoE^−/−^ mice. Given the known sex differences in the incidence and progression of atherosclerosis, the inclusion of female animals in future studies will be important to assess potential sex-specific effects of RA. Third, the absence of additional overexpression or knockdown techniques at the animal level to further confirm this pathway in vivo. To strengthen the findings and provide more definitive evidence, future studies should focus on using these techniques in animal models of AS. Moreover, although our results strongly suggest that AKR1B1 is a direct target of RA’s protective effects on endothelial cells, additional in vivo and in vitro experimental validation is necessary to conclusively establish this relationship. We also acknowledge that a healthy control group treated with RA alone was not included; while existing literature and our in vitro data support the safety of RA, this represents a limitation that should be addressed in future studies to further confirm its safety profile in non-diseased states. Further studies should aim to provide more direct evidence of RA’s interaction with AKR1B1 and its subsequent effects on the AKR1B1/SIRT3/PFKFB3 signaling axis. While our data collectively support a model wherein RA inhibits AKR1B1 to restore NAD^+^ homeostasis and subsequently activate the SIRT3/PFKFB3 axis, we acknowledge that direct genetic rescue experiments would provide further definitive evidence for this hierarchical relationship. Specifically, future studies employing SIRT3 or PFKFB3 knockdown in RA-treated cells, or SIRT3 overexpression in the context of AKR1B1 upregulation, would confirm whether these molecules are essential downstream effectors of RA’s protective effects. Such experiments represent an important direction for further elucidating the precise molecular architecture of this signaling pathway. Lastly, while RA has shown promising results in this study, it is essential to explore other potential AKR1B1 inhibitors that may offer similar or even more potent protective effects on ECs in the context of AS. This consideration, along with the other limitations discussed, should be addressed in future investigations to fully validate the therapeutic potential of targeting this axis.

## 5. Conclusions

In conclusion, our findings demonstrated that RA effectively attenuates tunica intima injury in ApoE^-/-^ mice with AS. In vitro studies confirmed RA’s significant protective effects against Ox-LDL-induced injury in HUVECs. The therapeutic mechanism of RA is attributed to its ability to enhance vascular endothelial cell energy metabolism by modulating the AKR1B1/SIRT3/PFKFB3 signaling axis. Specifically, RA directly binds to AKR1B1, regulating both its protein expression and enzymatic activity. Collectively, these findings identify RA as a promising candidate for the treatment of AS.

## Figures and Tables

**Table 1:** The involved database URLs in Bioinformatics analysis.

Order	Database Name	URL	Search Parameters	Date of Access
1	TCMSP	https://tcmsp-e.com/target.php?qt=655	a score cutoff of 20 and altered *p*-value of 0.05	From 1 November 2023 to 30 May 2024
2	BATMAN-TCM	http://bionet.ncpsb.org.cn/batman-tcm/#/home	a score cutoff of 20 and altered *p*-value of 0.05	
3	SwissTargetPrediction	http://www.swisstargetprediction.ch/	probability > 0	
4	UniProt	https://www.uniprot.org/	set the organism classification to “*Homo sapiens*”	
5	GeneCards	https://www.genecards.org/	/	
6	PharmGKB	https://www.pharmgkb.org/	/	
7	TTD	http://db.idrblab.net/ttd/	/	
8	DrugBank	https://go.drugbank.com/		
9	Venn platform	https://bioinfogp.cnb.csic.es/tools/venny/index.html	/	
10	STRING	https://cn.string-db.org/	a minimum interaction score of 0.90	

**Table 2:** Primer sequences used for RT-qPCR analysis.

Primer Name	Sequence
Human-β-actin-F	TGGCACCACACCTTCTACAA
Human-β-actin-R	CCAGAGGCGTACAGGGATAG
Human-AKR1B1-F	TGCCACCCATATCTCACTCA
Human-AKR1B1-R	TGTCACAGACTTGGGGATCA

**Table 3:** Common targets of RA, AS, and EC Protection.

Gene Names	Protein Names
IL5	Interleukin-5
CD80	T-lymphocyte activation antigen CD80
CD86	T-lymphocyte activation antigen CD86
CCL11	C-C motif chemokine 11
IDO1	Indoleamine 2,3-dioxygenase 1
C3	Complement C3
C5	Complement C5
AKR1B1	Aldo-keto reductase family 1 member B1
TTR	Transthyretin
MMP1	Matrix metalloproteinase-1
MMP9	Matrix metalloproteinase-9
MMP2	Matrix metalloproteinase-2
MMP12	Matrix metalloproteinase-12
MMP13	Matrix metalloproteinase-13
MMP3	Matrix metalloproteinase-3
AKR1B10	Aldo-keto reductase family 1 member B10
EGFR	Epidermal growth factor receptor
SELL	L-selectin
SELE	E-selectin
SELP	Selenoprotein P
PPARD	Peroxisome proliferator-activated receptor delta
MET	Hepatocyte growth factor receptor
IGFBP3	Insulin-like growth factor-binding protein 3
ALDH2	Aldehyde dehydrogenase
F3	Tissue factor
APP	Amyloid-beta precursor protein
ITGB1	Integrin beta-1
IFNG	Interferon gamma
PTGS1	Prostaglandin G/H synthase 1
ITGA4	Integrin alpha-4

## Data Availability

The original contributions presented in this study are included in the article/Supplementary Material. Further inquiries can be directed to the corresponding authors.

## Supplementary Materials

The following supporting information can be downloaded at: https://www.mdpi.com/article/10.3390/biom16030403/s1. Figure S1: The original WB images of Figure 3G,H; Figure S2: The original WB images of Figure 5C; Figure S3: The original WB images of Figure 7D; Figure S4: The original WB images of Figure 8B.

## Abbreviations

The following abbreviations are used in this manuscript:

AKR1B1	Aldo-keto reductase family 1, member B1
ANOVA	one-way analysis of variance
ApoE-/-	apolipoprotein-E-deficient
AS	atherosclerosis
ATP	adenosine 5-triphosphate
BCA	bicinchoninic acid assay
CCK-8	cell counting kit-8
CD31	platelet endothelial cell adhesion molecule-1
CETSA	cellular thermal shift assays
DAPI	4′,6-diamidino-2-phenylindole; DCFH-DA, 2′,7′-dichlorofluorescin diacetate
ECs	endothelial cells
ECAR,	extracellular acidification rate;
ECL	enhanced chemiluminescence
ECM	endothelial cell-specific culture medium
EP	epalrestat
ET-1	endothelin-1
F-6-P	β-D-fructose 6-phosphate
F-1,6-P	D-fructose 1,6-bisphosphate
G-6-P	D-glucose 6-phosphate
HFD	high-fat diet
HRP	horseradish peroxidase
HUVECs	human umbilical vein endothelial cells
KEGG	Kyoto Encyclopedia of Genes and Genomes
NAD+	nicotinamide adenine dinucleotide
NO	nitric oxide
OCR	oxygen consumption rate
ORO	Oil red O
Ox-LDL	oxidized low-density lipoprotein
PBS	phosphate balanced solution
PFKFB3	6-phosphofructo-2-kinase/fructose-2,6-biphosphatase 3
PPI	protein–protein interaction
P/S	penicillin-streptomycin;
PVDF	polyvinylidene difluoride
RA	rosmarinic acid
RIPA	radio-immunoprecipitation assay
ROS	reactive oxygen species
RT-qPCR	real-time quantitative PCR
SDS-PAGE	sodium dodecyl sulfate-polyacrylamide gel electrophoresis
SIRT3	Sirtuin 3
SPF	specific pathogen-free
3PG	3-phospho-D-glycerate
