# Supplementary material for: Rosmarinic Acid Targets AKR1B1 to Ameliorate Atherosclerosis via Vascular Endothelial Cell Energy Metabolism Regulation
Source: Biomolecules. 2026 Mar 9;16(3):403. doi: 10.3390/biom16030403 (PMC13023468; doi:10.3390/biom16030403)
Supplement: Supplementary file 1 [file biomolecules-16-00403-s001.zip › The original Western blot data.pdf]

The original Western blot data in the manuscript

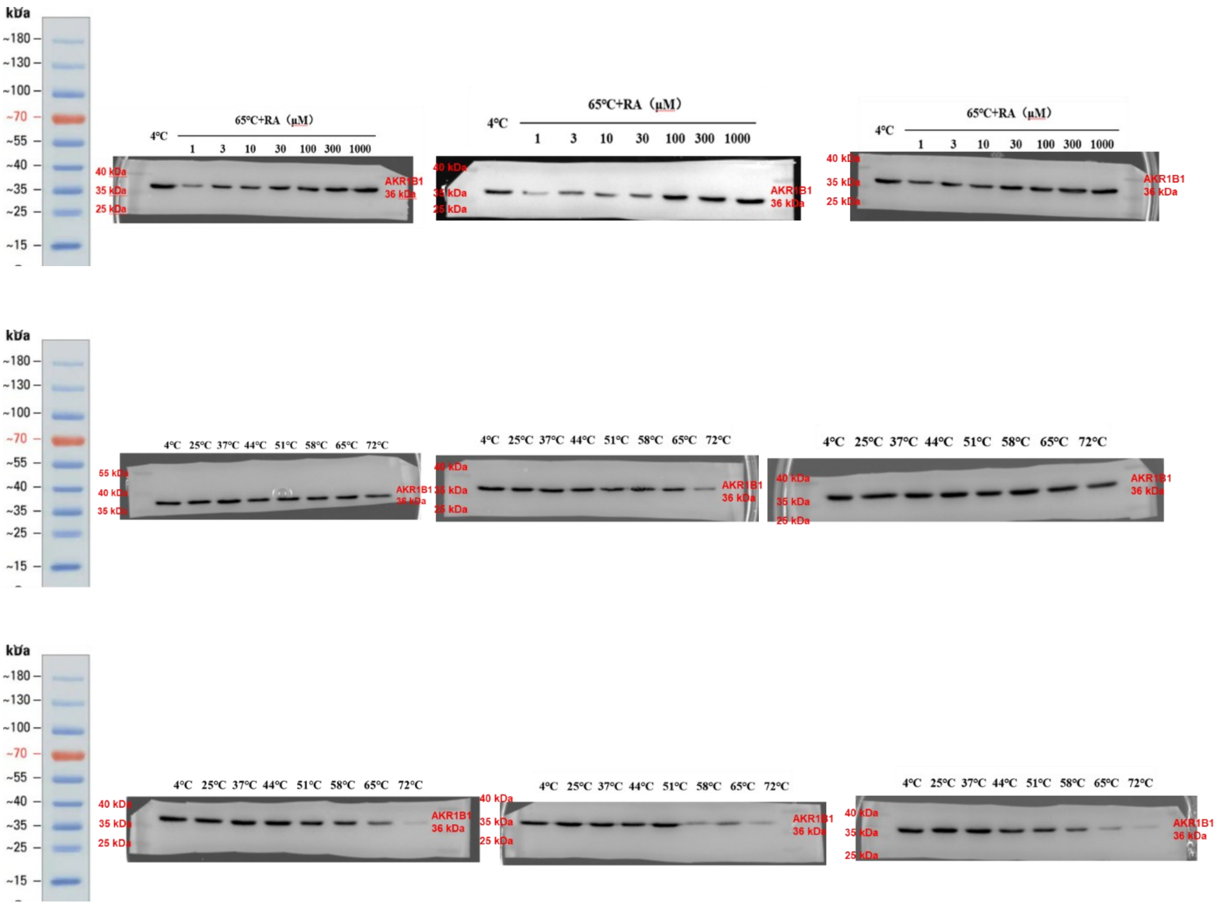

Figure S1 The original WB images of Figure 3G&H

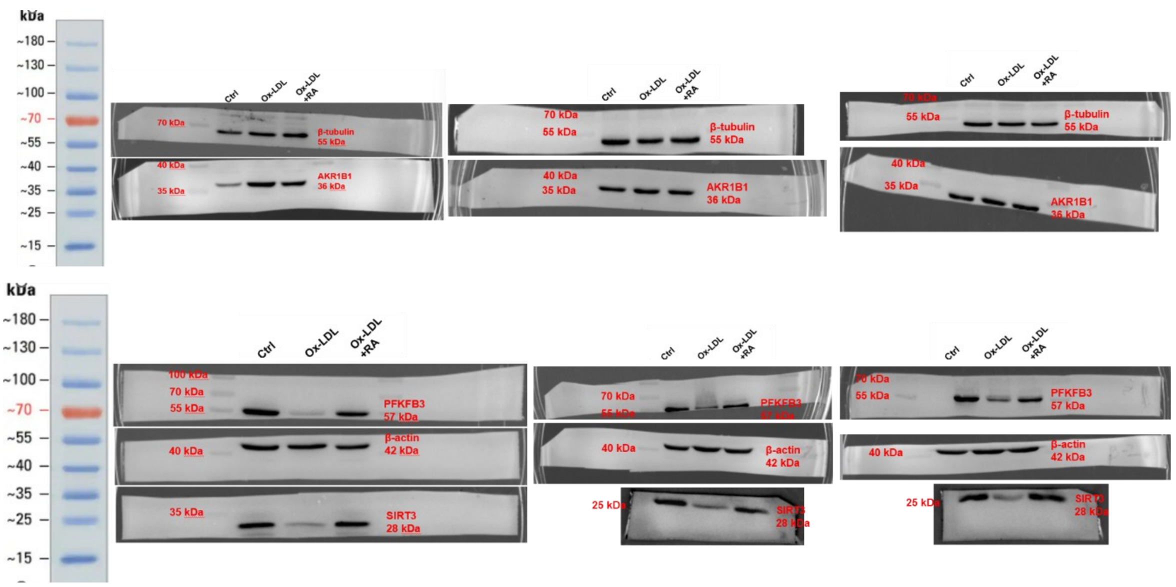

Figure S2 The original WB images of Figure 5C

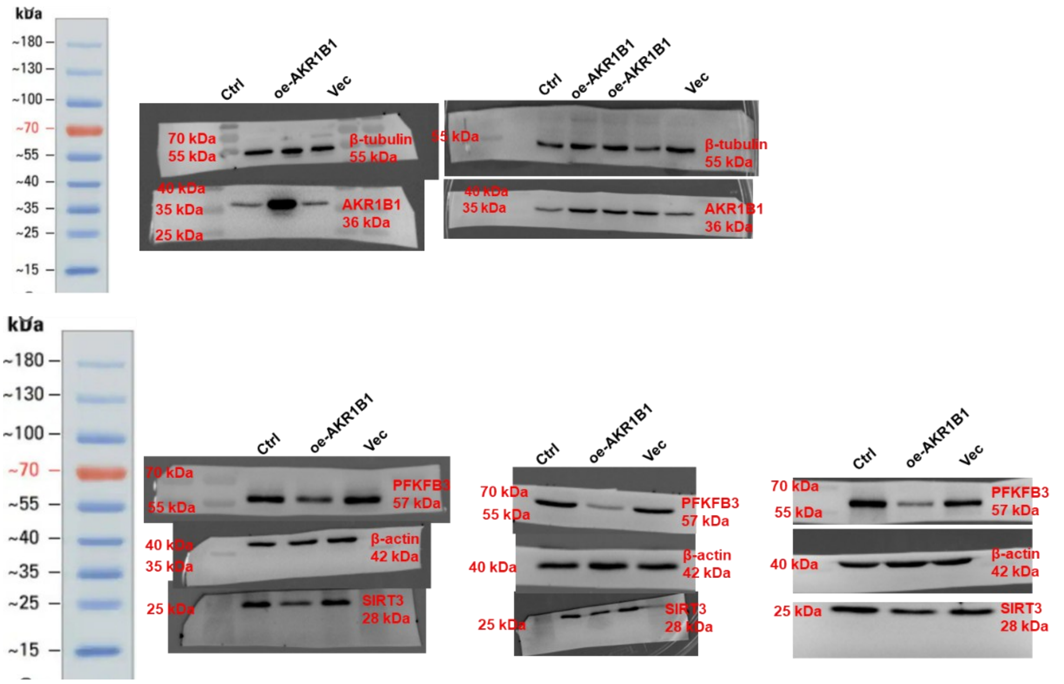

Figure S3 The original WB images of Figure 7D

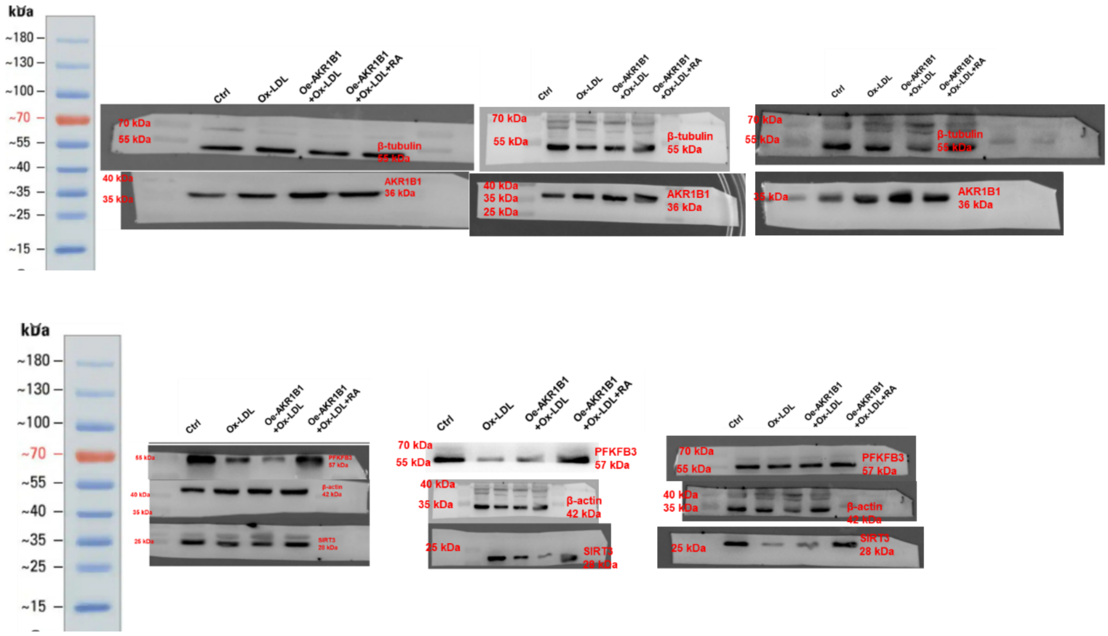

Figure S4 The original WB images of Figure 8B
